# COVID-19 pandemic impact on follow-up of child growth and development in Brazil

**DOI:** 10.3389/fped.2022.947493

**Published:** 2022-11-03

**Authors:** Lucas Lima Carneiro, Ed Wilson Rodrigues Vieira, Elysângela Dittz Duarte, Najara Barbosa da Rocha, Gustavo Velasquez-Melendez, Walmir Caminhas

**Affiliations:** ^1^ Department of Electrical Engineering, Universidade Federal de Minas Gerais, Belo Horizonte, Brazil; ^2^ Department of Maternal and Child Nursing and Public Health, School of Nursing, Universidade Federal de Minas Gerais, Belo Horizonte, Brazil; ^3^ Department of Community and Preventive Dentistry, School of Dentistry, Universidade Federal de Minas Gerais, Belo Horizonte, Brazil

**Keywords:** COVID-19, pandemics, health services accessibility, child care, growth and development

## Abstract

**Objectives:**

This study investigated the impact of the COVID-19 pandemic on the primary health care (PHC) services to follow-up the child growth and development (CGD) in Brazil.

**Methods:**

A cross-sectional study was conducted using secondary data related visits to assess the growth and development of children up to five years between Apr-2017 to Mar-2021. Differences between monthly rate of visits (per thousand inhabitants up to five) during the pandemic (Apr-2020 to Mar-2021) and before (Apr-2017 to Mar-2020) were analyzed using paired t test and control diagrams (averages ± 1.96 standard deviation).

**Results:**

A total of 39,599,313 visits for monitoring CGD was studied. The average monthly rate of visits dropped from 61.34 (per thousand) before the pandemic to 39.70 in the first 12 months of the pandemic (*p* < 0.001). In all states, except Rio Grande do Sul, there was a significant reduction, with differences ranging from −14.21% in São Paulo to −59.66% in Ceará. The Northeast region was the most impacted, being lower than expected in all 12 first months of pandemic.

**Conclusions:**

The number of visits to follow-up the CGD in PHC in Brazil decreased during the first year of the COVID-19 pandemic, varying over the months and between states and regions.

## Introduction

In February 2020, the first COVID-19 case was registered in Brazil, caused by the SARS-CoV-2 virus. Currently, in December 2021, there are more than 22 million cases and almost 616,000 deaths in the country (1). Besides the number of infected and dead people, there is the economic, social, cultural, political, and public health impact entailed by this situation.

Although the definitive impacts of the pandemic on health systems have not yet been revealed, in many countries, effects have been pointed out, with emphasis on the reduction in the use of health services for elective care, including a reduction in the rates of individual clinical care for children in primary care services (2–4). Visits to monitor the children's growth and development (CGD) were also undermined during the pandemic (5–7).

At first, both political-organizational and public health factors and individual decisions contributed to these impacts on health care services. From an individual point of view, the fear of contracting the disease may have been decisive in the intention of seeking care and, consequently, in the use of services (3, 4). From a political-organizational and public health point of view, measures to control the spread of contamination and ensure a response to the most serious cases converged to discourage the supply of routine and elective care, as well as its demand, including health care in programs focused on monitoring the CGD in primary health care services (PHC) (8, 9).

Monitoring the children's growth and development is part of one of the seven strategic axes of the National Policy for Comprehensive Child Health Care, in addition to being one of the actions that contribute to achieving global challenges such as the Sustainable Development Goals (10). In practice, it consists of periodic visits in which actions are carried out to promote health, breastfeeding, development, immunization, tracking of pathological conditions, prevention of accidents and monitoring of growth and body weight according to the children's age; and, in many situations, they favor access to diagnosis of both acute and chronic diseases.

Similar to other elective care in PHC, it is expected that services focused on monitoring the CGD has been reduced. Nevertheless, it is not yet known the size of this impact or its regional distribution after 12 months of the first case. In this sense, the objective of this study was to investigate the impact of the COVID-19 pandemic on the PHC services to follow-up the CGD in Brazil.

## Methods

This is a descriptive and analytical study, with an ecological cross-sectional design, using data from the Health Information System for Primary Care (SISAB, as per its Portuguese acronym), which belongs to the Brazilian Ministry of Health. SISAB has been mandatory throughout the country since June 2015 and is part of the e-SUS Primary Care (e-SUS AB, as per its Portuguese acronym) strategy (11).

Data about the monthly number of individual visits performed to assess the growth and development of children up to five years of age in PHC services throughout Brazil in the period from April 2017 to March 2021 were considered. Data extraction took place in June 2021 in an automated manner and directly from SISAB, through the process known as web scrapping or data scrapping (12). The extraction process was carried out using Node.js software, with code in *JavaScrip*t language to access the page https://sisab.saude.gov.br, fill in the forms according to a previously defined protocol and download reports referring to problems or conditions evaluated by health professionals in each of the months included in the studied period. Data from the Federal District for April, May and June 2017 were not available in the SISAB tool, and therefore were not studied.

Descriptive analyses were performed using absolute and relative frequencies of primary health care visits. The rates of visits for to assess the growth and development were calculated for each thousand children up to five years of age considering population estimates by age groups for Brazil, regions, states, and the Federal District (13).

Differences between the average rates of visits in the pre-pandemic periods (from April 2017 to March 2020) and during the pandemic (from April 2020 to March 2021) were calculated and compared using paired *t* test at a significance level of 5%.

Monthly visit rates in the first 12 months of the pandemic, month by month, were compared using control diagrams (14). The control diagrams were designed for each State, Federal District and Country region using averages of monthly pre-pandemic visit rates ± 1.96 standard deviation. This strategy allowed analyzing if pandemic visit rates were above or below historical limits.

In order to complement the analysis by States and the Federal District, Resultant Vectors Graphs (RVG) were used. It is a technique developed with the intention of synthesizing the information from the control diagrams in just one graph. Resultant Vectors Graphs include, simultaneously, three pieces of information in the diagrams: monthly visit rate above, within or below historical limits. From the diagrams, each time the visit rate exceeded the expected upper limit, a unit vector was assigned in the growth direction of the ordinate axis; when the rate was below the expected lower limit, a vector in the decreasing direction was assigned in the decreasing direction of the ordinate axis; and when the rate was within the expected limits, a unit vector was assigned in the growth direction of the abscissa axis. Finally, after “walking through” the entire diagram, the vectors were added to generate a resultant.

It is underlined that RVG can be composed of vectors resulting from more than one control diagram, with the resultant vectors displayed in a single figure. In RVG, if the vector is in the first quadrant, it will indicate an increase in the visit rate for the studied period; if it is in the fourth quadrant, the visit rate will have been lower than expected; and if it is close to the abscissa axis, the rate will be within historical limits.

All analysis were conducted using MATLAB software, version R2021a Update 4 (9.10.0.1710957), and its Statistics and Machine Learning Toolbox.

## Results

A total of 39,599,313 visits for monitoring CGD occurred between April 2017 and March 2021 were covered (Table 1). The proportion of cases studied during the pandemic ranged from 13.5% in the Northeast region to 21.8% in the South region. Among the states, it ranged from 11.9% in Ceará to 25.0% in Rio Grande do Sul.

**Table 1:** Number of visits before and during the pandemic, according to regions, states, and the Federal District.

Location	Number of studied visits (×1,000)^a^		
	Pre-Pandemic (Apr. 2017 – Mar. 2020)	During the pandemic (Apr. 2020 – Mar. 2021)	Total
**Brazil**	32,586.13 (82.29%)	7,013.18 (17.71%)	39,599.31
**Mid-West**	1,717.84 (81.64%)	386.29 (18.36%)	2,104.13
Distrito Federal	358.13 (79.28%)	93.59 (20.72%)	451.71
Goiás	583.12 (81.44%)	132.86 (18.56%)	715.98
Mato Grosso	484.82 (84.97%)	85.73 (15.03%)	570.54
Mato Grosso do Sul	291.77 (79.74%)	74.12 (20.26%)	365.90
**Northeast**	11,832.95 (86.45%)	1,854.22 (13.55%)	13,687.17
Alagoas	983.10 (86.86%)	148.68 (13.14%)	1,131.78
Bahia	2,465.78 (87.70%)	345.92 (12.30%)	2,811.70
Ceará	1,770.85 (88.12%)	238.74 (11.88%)	2,009.59
Maranhão	1,264.01 (82.56%)	266.92 (17.44%)	1,530.92
Paraíba	1,085.42 (86.96%)	162.71 (13.04%)	1,248.13
Pernambuco	2,290.60 (85.61%)	384.99 (14.39%)	2,675.60
Piauí	943.30 (86.71%)	144.55 (13.29%)	1,087.86
Rio Grande do Norte	698.58 (86.65%)	107.60 (13.35%)	806.18
Sergipe	331.30 (85.96%)	54.11 (14.04%)	385.41
**North**	2,880.28 (83.22%)	580.92 (16.78%)	3,461.20
Acre	77.78 (82.32%)	16.71 (17.68%)	94.49
Amapá	78.18 (82.41%)	16.69 (17.59%)	94.87
Amazonas	716.55 (81.14%)	166.54 (18.86%)	883.09
Pará	1,520.76 (83.94%)	290.90 (16.06%)	1,811.66
Rondônia	180.20 (84.96%)	31.91 (15.04%)	212.11
Roraima	72.69 (82.41%)	15.51 (17.59%)	88.21
Tocantins	234.12 (84.59%)	42.66 (15.41%)	276.78
**Southeast**	12,581.89 (79.75%)	3,194.09 (20.25%)	15,775.98
São Paulo	7,026.56 (77.91%)	1,992.16 (22.09%)	9,018.73
Espírito Santo	440.36 (82.52%)	93.28 (17.48%)	533.65
Minas Gerais	2,671.56 (80.42%)	650.43 (19.58%)	3,322.00
Rio de Janeiro	2,443.40 (84.21%)	458.21 (15.79%)	2,901.61
**South**	3,573.18 (78.17%)	997.66 (21.83%)	4,570.84
Paraná	1,339.47 (79.36%)	348.40 (20.64%)	1,687.87
Rio Grande do Sul	1,187.00 (75.00%)	395.59 (25.00%)	1,582.59
Santa Catarina	1,046.70 (80.49%)	253.67 (19.51%)	1,300.38

^a^ Visits to monitor the growth and development of children up to five years of age.

The average monthly rate of visits for monitoring children's growth and development in Brazil (per thousand children up to five years of age in the population) dropped from 61.34 before the pandemic to 39.70 in the first 12 months of the pandemic (*p* < 0.001), a drop of 35.28% (Table 2). In all states, except Rio Grande do Sul, there was a significant reduction in the monthly visit rate, with differences ranging from −14.21% in São Paulo to −59.66% in Ceará.

**Table 2:** Differences among the average rates of visits before and during the pandemic, according to regions, states, and the Federal District.

Location	Average rates (per 1,000 inhab.)^a^		Differences among the rates	Standard error	*p* ^b^
	Pre-Pandemic (Apr. 2017–Mar. 2020)	During the pandemic (Apr. 2020–Mar. 2021)			
**Brazil**	61.34	39.70	−21.64 (−35%)	3.09	<0.001
**Mid-West**	39.58	26.29	−13.29 (−34%)	1.78	<0.001
Distrito Federal	53.58	37.86	−15.73 (−29%)	3.71	0.001
Goiás	31.56	21.47	−10.09 (−32%)	1.37	<0.001
Mato Grosso	47.70	25.20	−22.49 (−47%)	2.04	<0.001
Mato Grosso do Sul	36.99	28.18	−8.81 (−24%)	2.17	0.002
**Northeast**	79.16	37.37	−41.78 (−53%)	4.06	<0.001
Alagoas	106.95	49.25	−57.69 (−54%)	6.87	<0.001
Bahia	66.63	28.13	−38.50 (−58%)	3.53	<0.001
Ceará	75.14	30.31	−44.82 (−60%)	4.11	<0.001
Maranhão	59.68	37.88	−21.80 (−37%)	3.28	<0.001
Paraíba	105.98	47.46	−58.52 (−55%)	6.33	<0.001
Pernambuco	91.75	46.89	−44.86 (−49%)	5.15	<0.001
Piauí	110.13	50.77	−59.36 (−54%)	5.03	<0.001
Rio Grande do Norte	80.78	37.75	−43.03 (−53%)	3.99	<0.001
Sergipe	54.18	26.57	−27.61 (−51%)	3.97	<0.001
**North**	49.57	30.07	−19.50 (−39%)	3.08	<0.001
Acre	25.82	16.77	−9.05 (−35%)	1.53	<0.001
Amapá	26.87	17.37	−9.49 (−35%)	1.98	0.001
Amazonas	48.85	34.36	−14.49 (−30%)	4.47	0.008
Pará	58.57	33.79	−24.78 (−42%)	3.85	<0.001
Rondônia	35.96	18.99	−16.97 (−47%)	1.52	<0.001
Roraima	35.62	21.59	−14.03 (−39%)	2.78	<0.001
Tocantins	52.05	28.28	−23.77 (−46%)	2.37	<0.001
**Southeast**	60.24	46.12	−14.12 (−23%)	3.27	0.001
São Paulo	63.79	54.73	−9.06 (−14%)	3.96	0.043
Espírito Santo	42.67	27.02	−15.65 (−37%)	3.07	<0.001
Minas Gerais	55.83	40.84	−14.99 (−27%)	2.45	<0.001
Rio de Janeiro	60.29	34.00	−26.29 (−44%)	4.12	<0.001
**South**	50.05	41.95	−8.10 (−16%)	2.80	0.015
Paraná	47.11	36.82	−10.29 (−22%)	2.45	0.002
Rio Grande do Sul	46.23	46.66	0.43 (1%)	3.06	0.891
Santa Catarina	60.55	43.43	−17.12 (−28%)	3.22	<0.001

^a^ Rate of visits to monitor the growth and development of children per thousand children up to 5 years of age in the population.

^b^ Paired *t* test.

**Figure 1:**
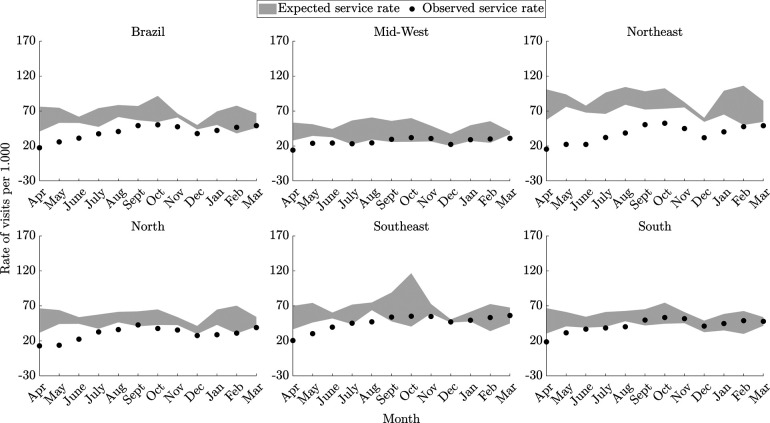
Monthly rates of visits (per thousand children up to five years of age) to monitor the growth and development of children, according to regions in primary health care services in Brazil. In gray: expected rates – pre-pandemic period (from April 2017 to March 2020); dots in black: observed rates – during the pandemic (from April 2020 to March 2021); the shaded areas indicate the average +/− 1.96 standard deviations for expected service rates.

The average monthly rate of visits in the Northeast region was the most impacted, being lower than expected in the 12 months of the first year of the pandemic. In the Mid-West, Southeast and South, the average monthly rate of visits was less impacted, being below those expected for 5 months during the first 12 of the pandemic period (Figure 1).

**Figure 2:**
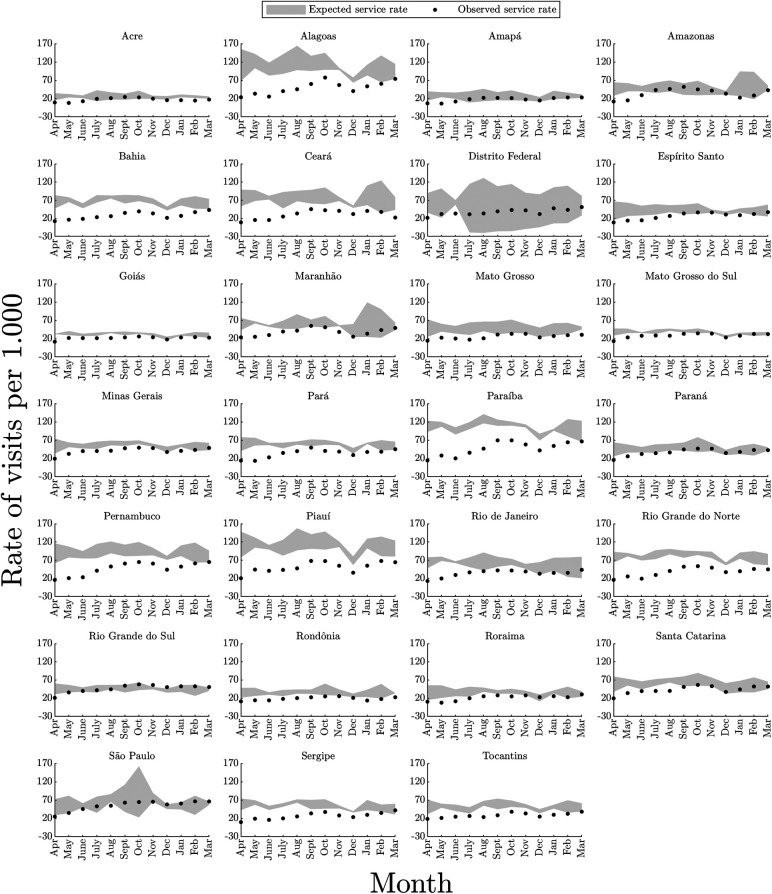
Monthly rates of visits performed in primary health care services, per thousand children up to five years of age, to monitor the growth and development of children, according to states and the Federal District in Brazil. In gray: expected rates – pre-pandemic period (from April 2017 to March 2020); dots in black: observed rates – during the pandemic (from April 2020 to March 2021); the shaded areas indicate the average +/− 1.96 standard deviations for expected service rates.

**Figure 3:**
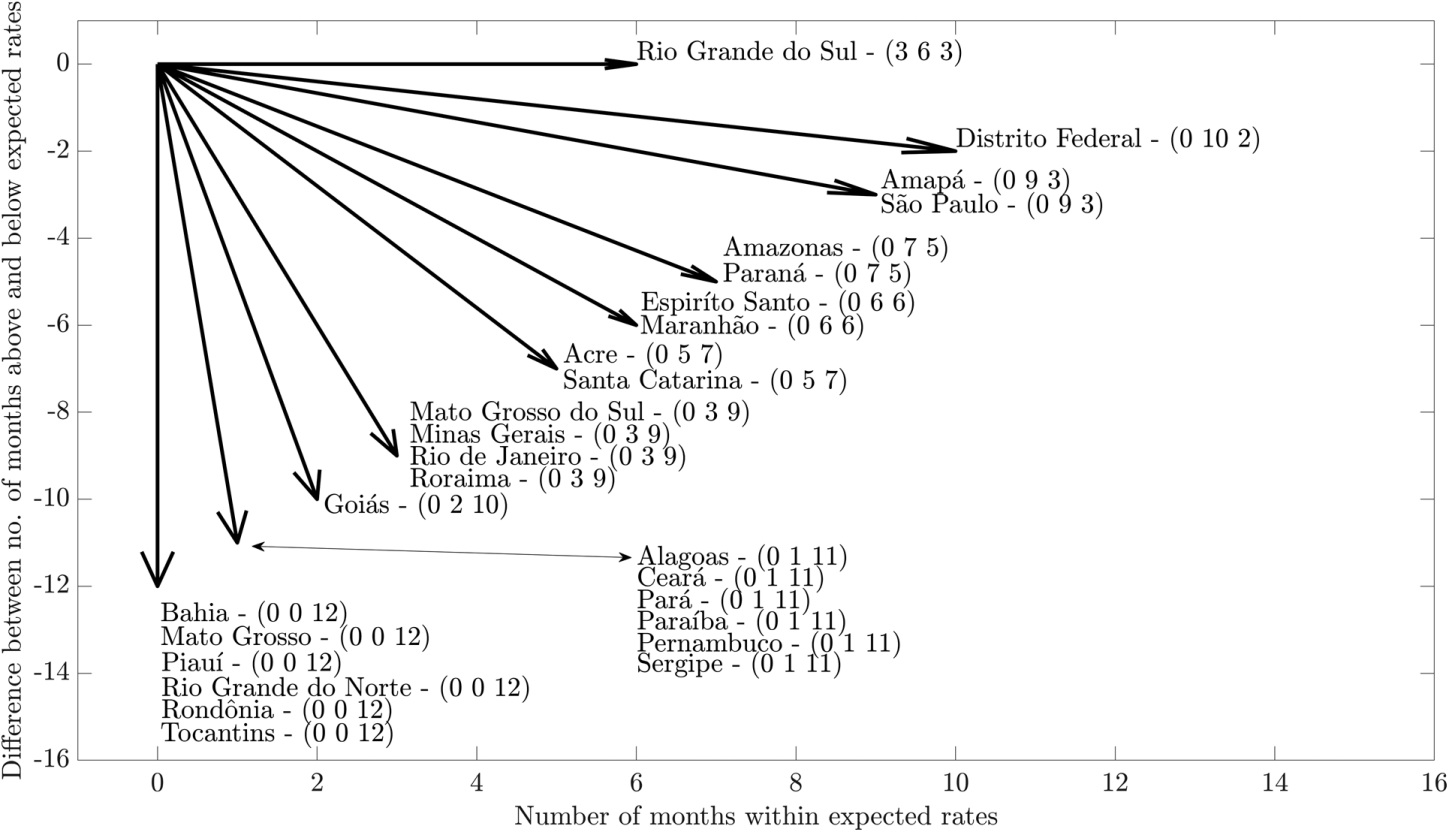
Resultant vectors obtained from the control diagrams of monthly rates of visits performed in primary health care services in Brazil, per thousand children up to five years of age, during the pandemic (from April 2020 to March 2021) compared to pre-pandemic period (from April 2017 to March 2020), according to states and the Federal District. Numbers in parenthesis represents number of months above, within and below expected rates, respectively.

Regarding monthly rates of visits for CGD by States and the Federal District, the greatest impacts were identified in those of the North region (Pará, Rondônia and Tocantins), Northeast (Alagoas, Bahia, Ceará, Paraíba, Pernambuco, Piauí, Rio Grande do Norte and Sergipe) and Mid-West (Mato Grosso do Sul), with visit rates below those expected for 11 months or more during the pandemic period. In Rio Grande do Sul, the smallest impact on rates was identified, with three months below expectations and three months above expectations. The Federal District, Amapá and São Paulo were also States where the rate of visits suffered less impact, showing two or three months with visits below expectations. It is worth highlighting that the most intense reductions in the number of visits were observed from April to June 2020 (Figures 2, 3).

## Discussion

During the 12 months of the first year of the COVID-19 pandemic, the use of PHC services to monitor the CGD was significantly lower than in previous years in Brazil. This reduction was not uniform and took place significantly in practically all regions and states.

Recognizing that some degree of reduction was expected, since the restriction of elective care was one of the measures to control the COVID-19 pandemic in Brazil, it is emphasized that our study advances by presenting important dimensions of this reduction, such as its geographic distribution, its different intensities and duration in the first year.

The average reduction in the number of visits in the first 12 months of the pandemic was similar to that found by other studies carried out with shorter periods (up to the first three months) (7, 15, 16). This is also corroborated by another study, where the reduction was more intense in the period from April to May 2020 (15). As for regional differences in the reduction of visits, it should be considered the interdependence and inseparability of political, economic, and geographic aspects among the Brazilian regions. With its large territorial extension, the regional differences and disparities in Brazil are often worsened by different forms of political-economic command and by the available health workforce. Still in this regard, a study conducted in Rwanda also identified regional differences in the reduction of visits for children during the pandemic. Nonetheless, in Brazil, the restriction measures and the installed capacity of health services vary from region to region, and one cannot disregard the number of COVID-19 cases in each area (17).

With respect to the number of months during which the number of visits was lower than expected before recovering, in many Brazilian states, it was found that the resumption was much slower than what was observed in other countries (7, 16, 18). Thus, it can be assumed that the flexibility of measures to control the pandemic was not immediately reflected in the resumption of the number of visits for CGD in Brazil. In places where resumption was faster, hybrid services, that is, face-to-face and virtual, constituted an important strategy (19).

Regular visits for monitoring children's growth and development, strongly recommended from the early 1980s as a Public Policy, were of great importance for the reduction of infant mortality in the country (20). In this sense, it can be assumed that the identified reduction in the number of visits may determine negative impacts on mortality indicators (21). Furthermore, these impacts may increase the already worrying regional inequalities in the mortality rates of children less than 5 years of age, since the North and Northeast regions persist with the highest rates in the country (20).

The reduction in these primary health care visits represent a barrier to diagnosing problems with child development and to carrying out early interventions when necessary. This occurs in a scenario in which the COVID-19 pandemic itself carries the potential to profoundly affect the development of children (6, 22). With the reduction, there could also be an increase in food insecurity, resulting in more cases of malnutrition and obesity (23, 24).

It is also necessary to consider that, for monitoring children's growth and development, the visits represent an opportunity for regular health care of children with chronic conditions. Accordingly, it is assumed that interruptions in monitoring may weaken the care of children with chronic conditions, since regular visits to health professionals allow the early identification of conditions that undermine care (25).

The context of the pandemic and the measures imposed by the health recommendations imposed new determinants and health conditions on children, with emphasis on the effects on mental health, longer exposure to screens and electronic games (26–28). With such a significant decline in monitoring of children, many of the unhealthy and risky conditions may not have received the care they would require. It is also important to highlight the increase in cases of violence against children during the pandemic, which, in a context of reduced access to regular visits, may not have been diagnosed, since an important part of the cases are diagnosed during routine visits not motivated by the acts themselves (29).

Among the lessons that can be drawn from the obtained results, there is the need for the Brazilian Unified Health System to be prepared to guarantee non-face-to-face monitoring when in a context that precludes physical proximity between children and PHC professionals. In general, the use of Telehealth was an important strategy to overcome the barriers of social distancing imposed by the pandemic and to favor children's access to routine care in many scenarios (30). However, in Brazil, it requires more investments in technological and human infrastructure for its implementation (29). In this pandemic, it is a fact that virtual services have gained an important boost and may have come to stay. It is now also necessary to take care of the training of professionals with a view to guaranteeing distance care and the development of safe strategies for childcare examinations, with emphasis on anthropometric measurements.

Among the limitations of this study, it is highlighted the inherent nature of research with secondary data, such as the fact that the analyzed data were not collected specifically to answer the research question. It should also be highlighted the fact that the analyses were carried out with data aggregated by States, Regions, and country, which does not allow for an assessment of any differences among municipalities. New studies that assess the impact of the pandemic at the level of municipal health systems will be important because many decisions during the pandemic were decentralized to municipalities. New studies that consider the fact of analyzing the impacts on CGD from the perspective of socioeconomic differences will also be important, since the regional differences found in our study may have influenced these inequalities (19).

In conclusion, the COVID-19 pandemic appears to have represented a barrier in relation to access to visits in the scope of monitoring the growth and development of children less than 5 years of age in PHC services in Brazil, with geographically and temporally unequal impacts. Although the restriction of elective care in PHC was considered necessary to minimize the risk of transmission of COVID-19, the impact of these restrictions on children's health may be long-lasting. The resumption of services related to CGD by PHC professionals is an urgent need. For this purpose, it is recommended to reduce barriers to these primary health care visits and to adopt innovative solutions with the use of technologies.

## Data Availability

Publicly available datasets were analyzed in this study. This data can be found here: https://sisab.saude.gov.br/paginas/acessoRestrito/relatorio/estado/saude/RelSauProducao.xhtml.
